# Early clinical cxperience with the SIGN hip construct: a retrospective case series

**DOI:** 10.1051/sicotj/2018050

**Published:** 2016-11-30

**Authors:** Justin Roth, Brian Goldman, Lewis Zirkle, John Schlechter, John Ibrahim, David Shearer

**Affiliations:** 1 Department of Orthopaedic Surgery, Washington University, Campus Box 8233, One Children's Place, Saint Louis MO 63110 USA; 2 Largo Medical Center, 201 14th St SW, Largo, FL 33770 USA; 3 SIGN Fracture Care International, 451 Hills St #B, Richland, WA 99354 USA; 4 Adult and Pediatric Orthopedic Specialists, 1310 W Stewart Dr #508, Orange, CA 92868 USA; 5 University of California San Francisco, Orthopaedic Trauma Institute, 2550 23rd Street Building 9, 2nd Floor, San Francisco, CA 94110 USA

**Keywords:** Hip fracture, SIGN hip construct, Low- and middle income countries (LMICs), SIGN Online Surgical Database (SOSD), SIGN Fracture Care International.

## Abstract

*Background*: As the population ages, the developing world industrializes, and more urban centers emerge, the burden of orthopedic trauma will steadily increase. SIGN Fracture Care International has developed a unique intramedullary device for fixation of hip fractures in low-resource settings lacking fluoroscopy. The purpose of this study is to report the safety profile and complication rate for a consecutive series of hip fracture patients managed using this implant.

*Methods*: We conducted a retrospective analysis of the first 170 patients treated with the SIGN Hip Construct (SHC) from 2009 to 2014 using the SIGN Online Surgical Database (SOSD). Patients with follow-up greater than 12 weeks and adequate radiographs were included. Data recorded include patient demographics, time-to-surgery, union rate, AO/OTA classification, complications, neck-shaft angle, and clinical outcomes including painless weight bearing and knee flexion greater than 90°.

*Results*: Of 170 patients, 71 met inclusion criteria with mean follow-up of 39 weeks. Mean age was 49.5 and by WHO, regions were Africa (27), Eastern Mediterranean (21), Western Pacific (17), Americas (3), and Southeast Asia (3). Fractures included intertrochanteric (55), subtrochanteric (7), femoral neck (4), and combined (5). Reduction quality was good in 35 (49%), acceptable in 19 (27%), and poor in 17 (24%). Major complications consisted of varus collapse (6), non- or delayed union (3), intra-articular screw (5), and infection (3). Average postoperative neck-shaft angle was 126° and 119.3° at final follow-up.

*Conclusions*: This is the first comprehensive report of a novel implant for hip fractures specifically designed for low-resource settings. The early clinical data and outcomes suggest that the SHC can be safely inserted in the absence of fluoroscopy, and facilitates early mobilization while maintaining acceptable reduction until union.

## Introduction

As the global population grows and ages, the number of hip fractures increases annually. Hip fractures totaled 1.6 million in 1990 and are projected to reach 6.26 million by 2050 [1]. Developing countries in Asia, Africa, and Latin America are predicted to experience the largest growth due to the aging population and increasing prevalence of osteoporosis [2]. Hip fractures most often result from low-energy falls in older patients or less commonly from high-energy trauma in younger patients [3]. These fractures are typically stabilized with either a sliding hip screw or a cephalomedullary nail with the latter preferred for unstable patterns [4–6]. The need for fluoroscopy for safe insertion and high cost of these implants are substantial barriers to treating hip fractures in low-income countries [7].

**Figure 1:**
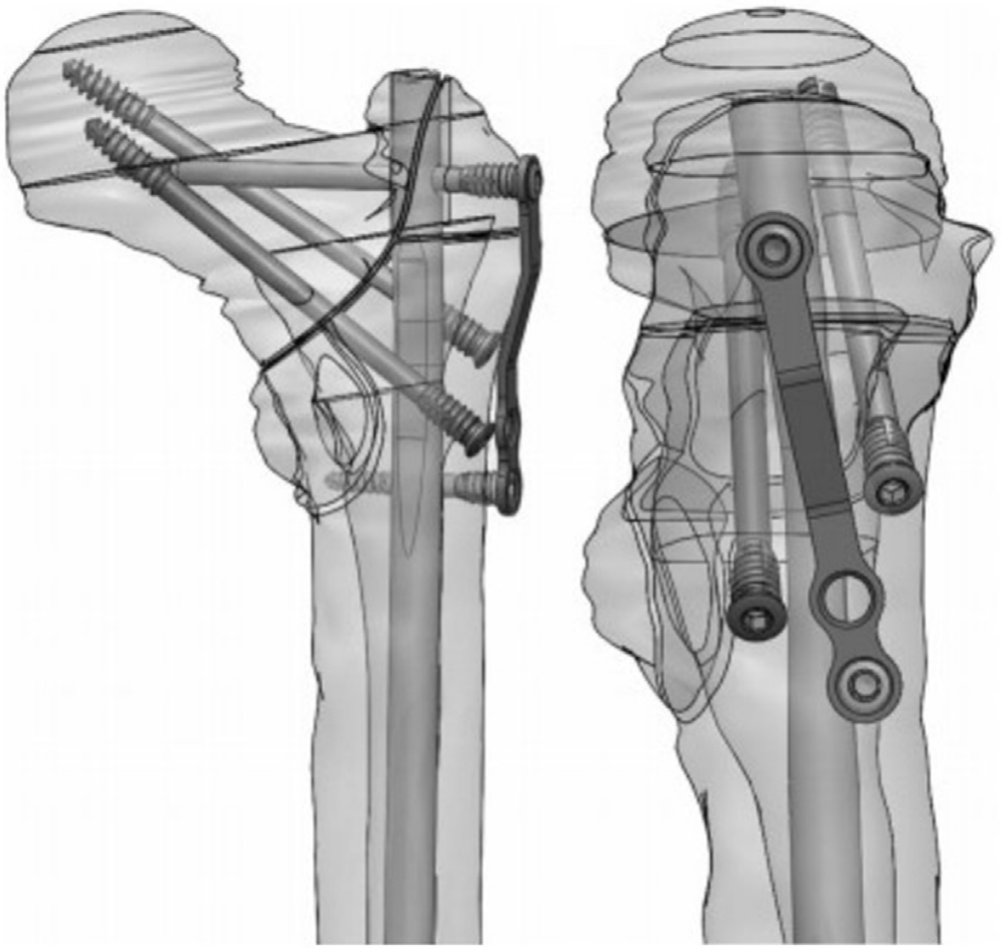
SIGN hip construct (SHC) design.

The SIGN Hip Construct (SHC) was designed to overcome these barriers (Figure 1). The SHC uses simple hand instruments combined with an open reduction to diminish the need for fluoroscopy [8]. Implants are donated at no cost to participating hospitals provided sites provide surgery and follow-up data through an online database [9–13]. At the time of writing, 47 hospitals are using the SHC globally and more sites are being established through on-site training. However, limited data have been published reporting outcomes of treatment with this novel implant.

This study aims to evaluate patient demographics, safety, and complications in the first series of patients treated with the SHC for proximal femur fractures. Secondary outcomes include clinical and radiographic union, alignment, and range-of-motion. We hypothesize that the SHC provides stable fixation of proximal femur fractures resulting in union and complication rates similar to historical controls.

## Materials and methods

**Figure 2:**
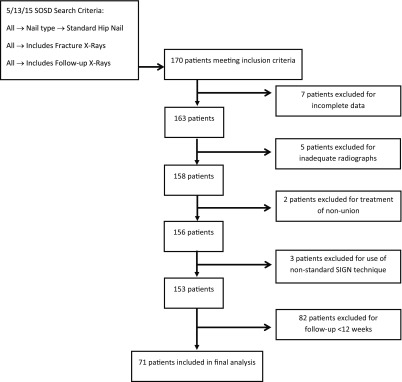
Patient selection flowchart.

We conducted a retrospective analysis of the SIGN Online Surgical Database (SOSD). The SOSD is a prospective registry maintained by the implant manufacturer. Approval for use of the SOSD was provided in April of 2015 by SIGN Fracture Care International. Participation in the database is mandatory for hospitals to continue receiving donated implants. After each case, the surgeon must upload demographic data, injury characteristics, surgical treatment, and pre-, postop, and follow-up radiographs. Data were extracted from the SOSD on May 13, 2015, for 2009–2014 using the following inclusion criteria (Figure 2): standard hip construct nail, fracture radiographs available, and follow-up radiographs available.

There were 170 patients meeting these criteria. These cases were subsequently reviewed manually and excluded using the following criteria: incomplete data, inadequate radiographs, primary diagnosis of nonunion (surgeon defined), nonstandard technique (e.g., use of only compression screws or only IM nail), and follow-up <12 weeks. Our final analysis included 71 patients.

The SHC (SIGN Fracture Care International, Richland, WA) is a cephalomedullary device available in lengths ranging from 280 to 320 mm. The medial to lateral nail bend is 6° proximally, which allows for insertion through the tip of the greater trochanter and thus direct access to the femoral canal. Fracture fixation and compression are achieved using a single proximal interlocking screw and two compression screws inserted along the anterior and posterior walls of the greater trochanter into the femoral head. Distal fixation consists of either the fin nail (patented distal tapered spline) or static interlocking screws placed via the SIGN technique and target arm without fluoroscopy. The surgical technique has previously been described along with the methodology behind the device design [8].

A template is used with the preoperative X-rays to estimate the length of the compression screws, interlocking screw, and approximate angle of the compression screws. At the time of surgery, the patient is placed in the lateral decubitus position on a standard operating table. An incision is made over the lateral aspect of the greater trochanter starting 1 cm proximal to the vastus ridge extending to 4 cm below. Reduction is accomplished by traction and rotation and assessed by palpation or direct visualization through an anterolateral exposure. Clamps can be used to maintain the reduction, taking care to avoid blocking the insertion site for the compression screws. Additional compression and provisional fixation are accomplished by placing a compression screw anterior to the anticipated nail path into the femoral head. Insertion of the anterior compression screw begins with a hole made in the cortex 1 finger breadth below the vastus ridge in-line with the anterior femoral canal. A hand drill, known as the pilot drill, is used to make the track for the compression screw. The pilot drill provides greater tactile feedback than a typical power drill and is analogous to a pedicle finder used in the spine. To assist in matching the angle determined from the preoperative templating, an angle guide, known as the Z angle finder, can be used, which has a 130° angle relative to the lateral cortex. After the compression screw is seated, the start site for the nail is made through a separate incision at the tip of the greater trochanter at the junction between the posterior and middle third. The start site is determined by palpation with the finger and created using an awl. If needed, sequential reaming can be performed using hand reamers. The nail is then inserted and oriented such that the external jig directs the proximal interlocking screw into the femoral head. Using the target arm, the distal interlock is placed first, followed by the proximal interlock, using the technique previously described for the SIGN Intramedullary Nail System [13]. A second compression screw can be placed posterior to the nail into the femoral head. The entry hole for the posterior compression screw is made one finger-breadth distal to the anterior compression screw using the Z angle finder and the pilot drill. There is an optional plate available that can be used to provide additional stability in the presence of a fracture of the lateral wall.

The data were primarily extracted from the SOSD. We considered age, sex, country, and injury characteristics (e.g., fracture pattern) to be the predictor variables. The outcomes extracted were complications, time to painless full weight bearing, radiographic fracture union, and time to knee flexion >90°. Fracture union, painless weight bearing, and knee motion were all assessed by the individual surgeons at each site entering data into the SOSD. However, to minimize bias, all radiographs were reviewed independently by a PGY5 orthopedic resident (J.R.) and verified by a fellowship-trained orthopedic traumatologist (D.S.). Additionally, preoperative, postoperative, and follow-up radiographs allowed measurement of the neck shaft angle using standard technique [14]. The number of compression screws, presence of lateral plate, and type of distal fixation were also recorded. On the postoperative radiographs, the quality of fracture reduction was graded as good (<5° varus/valgus), acceptable (5–10° varus/valgus), or poor (>10° varus/valgus) in reference to a normal of 130° [15]. The fracture was subjectively considered united based on callus formation, interval maintenance of fracture reduction, and disappearance of the fracture line [16]. Union and implant-related complications were assessed on the follow-up radiographs and through case notes entered by the SIGN surgeon into the SOSD. Nonunion was defined as lack of radiographic healing at 9 months postoperatively without healing progress over the previous 3 months.

Data were initially collected in Microsoft Excel (Redmond, WA) before being transferred to Stata 13.0 (College Station, TX) for analysis [17]. Descriptive statistics were calculated for demographic data, injury characteristics, and complications. Univariate analysis was conducted to assess for risk factors for complications using Fisher's exact test and significance set at *p* < 0.05. Paired student's *t*-test was used to compare changes in neck-shaft angle from baseline to follow-up.

## Results

Seventy-one patients were analyzed, 48 males and 23 females, with a mean age of 49.5 years (range 12–91). Mean follow-up was 39 weeks (range 21–64) (Table 1). The patients came from a wide array of WHO regions including Africa (27), Eastern Mediterranean (21), Western Pacific (17), Americas (3), and Southeast Asia (3). In total, 13 countries were classified as low or middle income (Table 1).

**Table 1:** Patient characteristics.

Age, mean (range)		49.5 (12–91)
Gender, No. (%)		
	Male	48 (67.6)
	Female	23 (32.4)
Follow-up in weeks, mean (range)		39 (21–64)
WHO region, No. (%)		
	Africa (Cameroon, Somaliland, Ethiopia, Tanzania, Kenya)	27 (38)
	Eastern Mediterranean (Pakistan)	21 (29.6)
	Western Pacific (Cambodia, Mongolia, Philippines)	17 (23.9)
	Americas (Dominican, Haiti, Nicaragua)	3 (4.2)
	Southeast Asia (Bangladesh)	3 (4.2)
Representation by country^a^, No. (%)		
	Pakistan	21 (29.6%)
	Ethiopia	15 (21.1%)
	Mongolia	10 (14.1%)
	Tanzania	8 (11.3%)
	Philippines	4 (5.6%)
	Bangladesh	3 (4.2%)
	Cambodia	3 (4.2%)
	Cameroon	2 (2.8%)
	Kenya	1 (1.4%)
	Dominican	1 (1.4%)
	Haiti	1 (1.4%)
	Nicaragua	1 (1.4%)
	Somaliland	1 (1.4%)
Open fracture, No. (%)		
	No	70 (98.6%)
	Yes	1 (1.4%)
Injury location, No. (%)		
	Intertrochanteric	55 (77.5%)
	Subtrochanteric	7 (9.9%)
	Intertrochanteric + subtrochanteric	4 (5.6%)
	Femoral neck	4 (5.6%)
	Femoral neck + intertrochanteric	1 (1.4%)
Days from injury to surgery, median (IQ range)		10 (5–21)
Lateral wall plate, No. (%)		
	Yes	29 (41%)
	No	42 (59%)
Distal fixation, No. (%)		
	Screw	66 (93%)
	Fin	1 (1.4%)
	None	1 (1.4%)
	Unknown	3 (4.2%)

a Countries represented are all low or middle income per WHO classification.

The majority of the fractures were closed (70) and the pattern was intertrochanteric (55). Subtrochanteric (7) and femoral neck (4) fractures were also included in addition to several combination fractures resulting from high-energy mechanisms: intertrochanteric + subtrochanteric (4) and intertrochanteric + femoral neck (1) (Table 1). The median time from injury to surgery was 10 days (range 5–21). A supplemental lateral wall plate was utilized in 29 cases (41%) and distal fixation in the form of an interlocking screw was used in 66 (93%) (Table 1).

Fracture reduction was good in 35 (49%), acceptable in 19 (27%), and poor in 17 (24%). A paired student's *t*-test comparing changes in neck-shaft angle is summarized in Table 2. Neck-shaft angle averaged 126° postoperatively and 119° at final follow-up with a difference of 6.9° (*p* < 0.0001). Immediate postoperative neck-shaft angle did not correlate with delay to surgery or subsequent varus collapse.

**Table 2:** Neck-shaft angle.

Immediate post-op	126° ± 7.3°
Final follow-up	119.3°± 11°
Difference	−6.9° ± 8.2°*

*p = <0.0001.

Major complications included varus collapse >15° (six patients), intra-articular screw (five), nonunion (three), and deep infection (one) (Table 3). Fisher's exact test was used to determine the association of risk factors on complications. The single independent risk factor associated with varus collapse >15° was age ≥50 years (*p* = 0.027). Gender, fracture location, delay to surgery, open fracture, postop neck-shaft angle, and type of SHC (lateral plate, distal fixation) were not associated with varus collapse. The most significant risk factor for intra-articular screw placement was the SIGN site with four of five cases coming from a single center (*p* < 0.0001). Additionally, patients with intra-articular screw placement tended to be older with a mean age 65 versus 48 (*p* = 0.08).

**Table 3:** Major complications.

Complication	No. (%)
Varus collapse (>15⁰)	6 (8.5%)
Nonunion/delayed union	3 (4.2%)
Intra-articular screw	5 (7%)
Infection (deep)	1 (1.4%)

Subjective assessments by the SIGN surgeon included a mean time to painless full weight bearing at 32.8 weeks and mean time to knee flexion >90° at 25 weeks. At final follow-up, 95.8% of patients achieved union and 90.1% of patients attained full painless weight bearing. Knee flexion of at least 90° was achieved in 98.6% of cases.

## Discussion

Proximal femoral fracture fixation, despite being a common orthopedic surgery, remains an unsolved problem in the developing world where many patients are still treated in traction with prolonged nonweight bearing. This study is the first report of an initial series of patients treated using the SIGN Hip Construct in 13 low- or middle-income countries on three continents. We hypothesized that the SHC would provide stable fixation of proximal femur fractures with acceptable union and complication rates similar to historical controls.

Limitations of our study include its retrospective nature, lack of a comparison group, irregular patient follow-up intervals, and 50% loss to follow-up. Further limitations associated with use of the SOSD were low quality of the radiographic images, lack of case-specific details, and self-reported clinical outcomes from surgeons. Despite these limitations, our study uniquely reports the outcomes of a novel implant used in a low-resource setting with a relatively large sample size and greater than 12-week follow-up.

The safety profile and complication rates associated with the SHC are of paramount concern because the technique is an open reduction that avoids image intensification and thus relies heavily on the skill and operative technique of the surgeon. Overall, the major complication rate was moderate. Complications included varus collapse, intra-articular screw placement, nonunion or delayed union, and infection.

**Figure 3:**
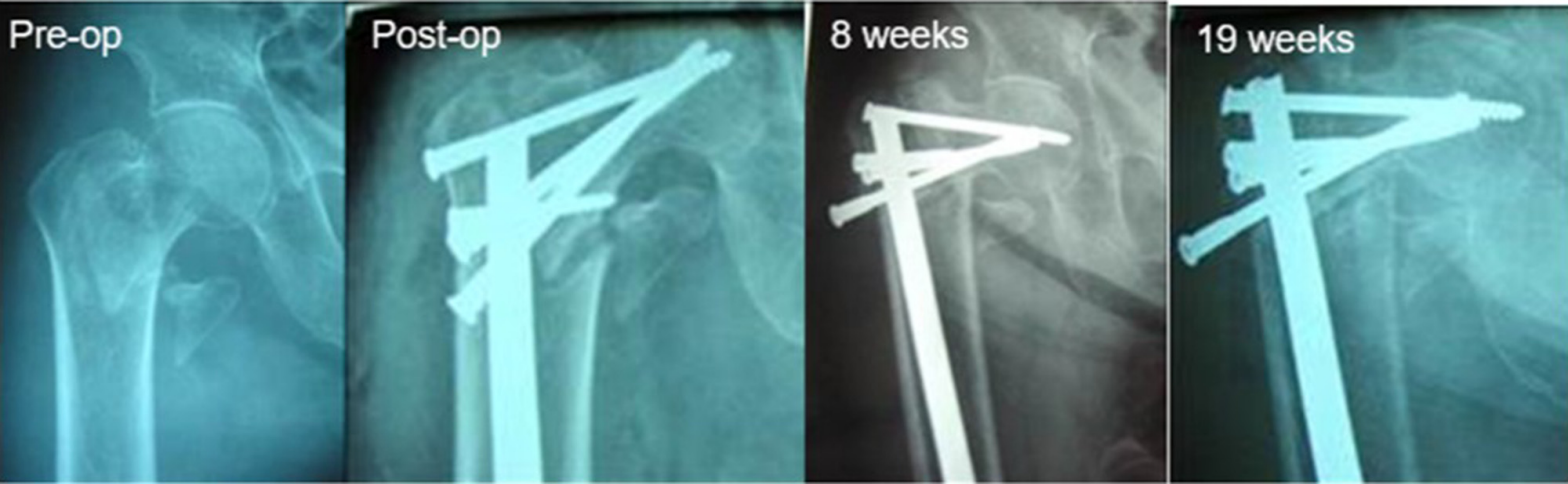
Radiographs of an 80 year old woman who experienced varus collapse but had painless full weight bearing at 8 weeks after surgery.

Our rate of varus collapse (Figure 3) of 8.5% is consistent with the 11.8% rate of collapse reported by Haonga et al. [2]. Of the six patients, four achieved painless weight bearing and two were lost to follow-up. Five of six patients suffering varus collapse had significant displacement preoperatively and all were unstable patterns (31A2.2(3), 31A3.3(1), and 31-B2(1)). The single stable pattern (A1.1) was a IIIb open injury that subsequently became superficially infected, eventually resolved, and proceeded to a delayed union. Interestingly, two of six constructs that went into varus collapse were placed higher on the greater trochanter than suggested, with fixation crossing only the superior third of the femoral neck, possibly contributing to construct failure with fixation only on the tension side of the neck and a lack of calcar stability. The single risk factor associated with varus collapse was advanced age (>50), suggesting bone quality likely played a role in construct failure. There was no association between varus collapse and delayed time to surgery, potentially because the number of patients treated at 4 weeks was too small and/or the 15° varus collapse threshold was too high.

**Figure 4:**
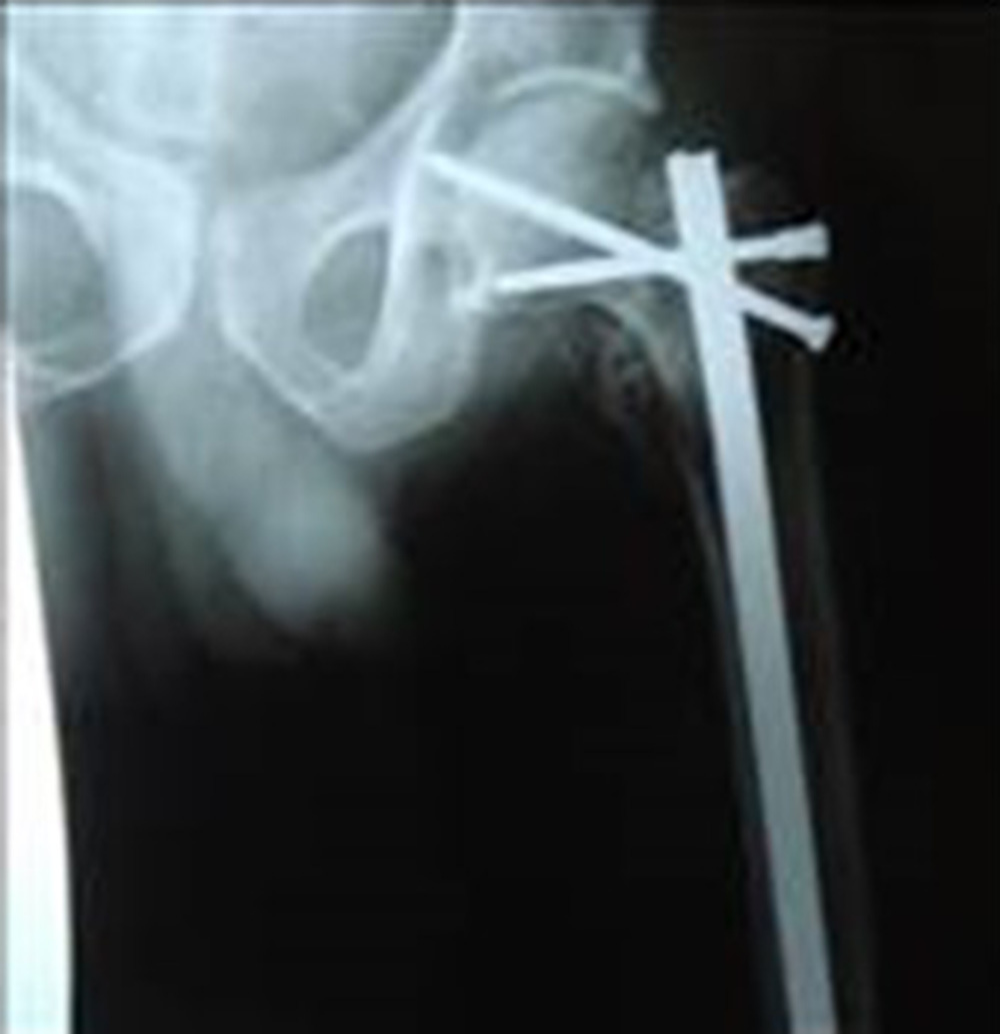
Intra-articular screw placement.

Five patients had intra-articular screw placement noted on postop X-rays (Figure 4), but four of the five patients were operated on at a single center, suggesting that further training and experience may reduce the complication rate. Surgeons, particularly those inexperienced with the technique, should move the hip through a range-of-motion prior to closing and consider an intra-operative plain AP X-ray. At final follow-up, all intra-articular screws had been removed. All patients with an intra-articular screw were older, suggesting poor bone quality as a risk factor for screw malposition.

**Figure 5:**
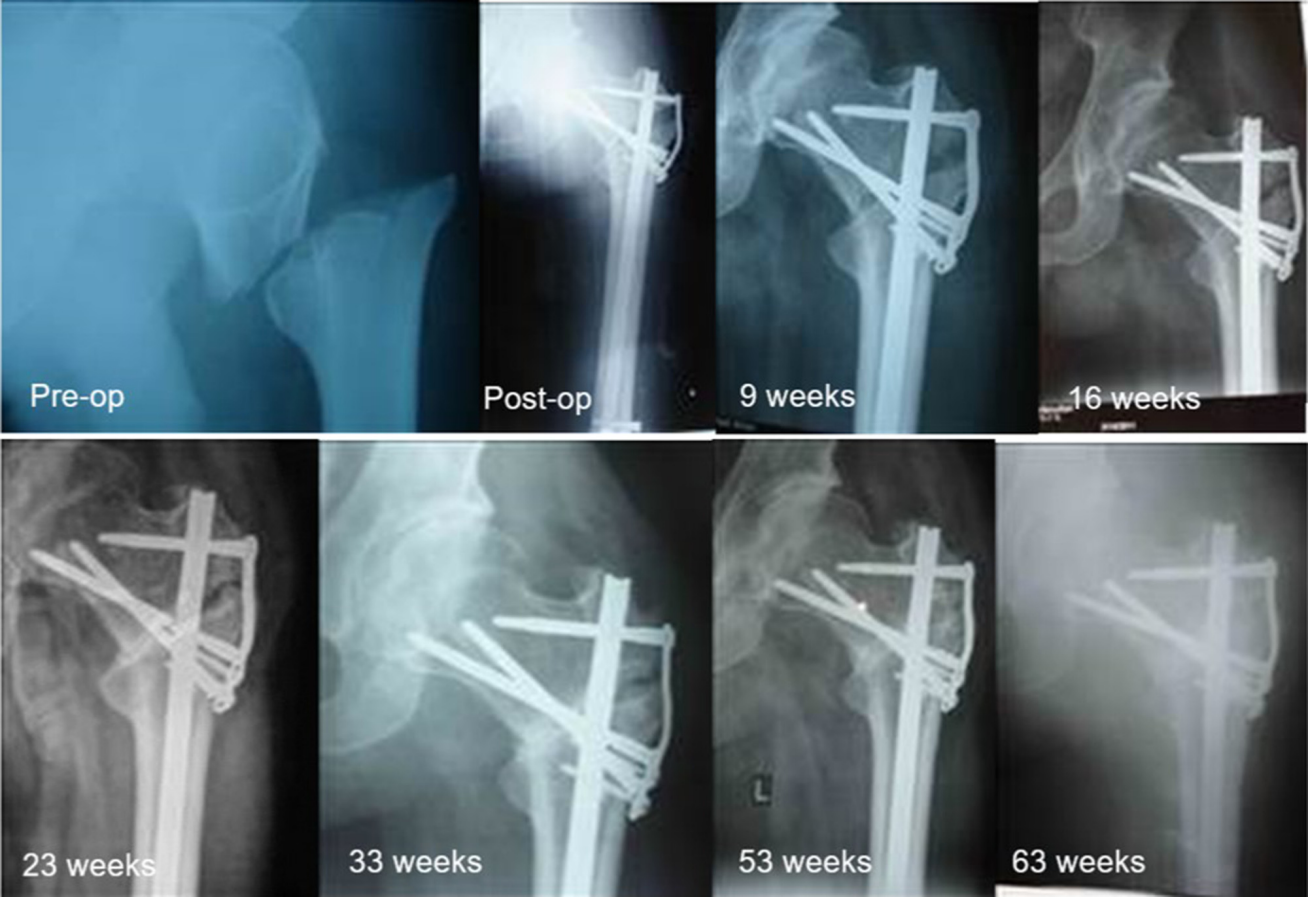
Radiographs of a 30 year old man with delayed union at 33 weeks after surgery but had painless full weight bearing at 9 weeks after surgery.

Nonunion was noted in two patients (2.8%) and delayed in union in one patient (1.4%) with their last radiographs recorded at weeks 62, 40, and 19, respectively. The published nonunion rates for intertrochanteric fractures range from 1–2% for operative treatment with fixation failure reported in up to 20% of unstable patterns [18]. The primary mode of failure was cutout, and initial fracture displacement was a major risk factor [18]. While there was a mean difference of −6.9° between pre- and final neck-shaft angle for the SHC (versus −2° by Mereddy et al. for the CMN [14]), it is clear that the SHC is stable enough to hold reduction even in a delayed union situation (Figure 5). Perhaps more importantly, no cases of screw cutout after insertion led to hip arthritis. This is particularly relevant in centers lacking access to total hip arthroplasty and therefore lack a salvage for screw cutout. A varus malunion may be a much more functional long-term outcome than cutout of a large-diameter lag screw in this setting.

Deep infection was noted in one patient (1.4%). This is similar to the 1.5% infection rate found by Haonga et al. in 68 patients treated with the SHC in Tanzania [2]. As a comparison, other authors have shown deep or periprosthetic infection rates with CMN, DHS, and intramedullary nailing to be between 0–3.2% [14,19,20]. The low rate is likely because of the generous soft tissue envelope and low open fracture rate [21].

Reduction quality in the absence of fluoroscopy was good/acceptable in 76% of cases. Comparatively, Mereddy et al. showed a good/acceptable rate of 97% for fluoroscopically guided CMN fixation utilizing the same criteria [14]. While fluoroscopy can aid reduction quality, without contralateral comparison neck-shaft angles as a control, it is impossible to discern how variable patient anatomy skewed our assessment. For this reason, if a single radiograph is to be obtained, we recommend an AP pelvis including both hips as opposed to only the injured side. Poor reduction quality may also contribute to varus collapse, though data did not support this association.

Time to union and fracture healing were difficult to assess because follow-up intervals were inconsistent and large gaps in time existed between radiographs. We therefore reported the union rate at final follow-up at 95.8% rather than time to union. Mereddy et al. reported a similar union rate of 92% at 12–16 weeks with four taking 24–32 weeks[14].

To the best of our knowledge, this is the first comprehensive multicenter study evaluating the SIGN Hip Construct for treatment of proximal femoral fractures without fluoroscopy. We found an overall complication rate of 21.1% with nonunion (4.2%) and deep infection rates (1.4%) comparable to other devices. The rate of varus collapse was higher than in series using a CMN, which raises concern about the stability of the construct. However, 96% went on to radiographic union despite collapse and there were no cases of screw cutout provided the screws were not placed in the joint at the time of surgery.

## Conclusion

Proximal femoral fractures were treated successfully with the SHC in this series. Although radiographic outcomes and intra-articular screw penetration are concerns, preliminary data suggest that the SHC can be safely inserted in the absence of fluoroscopy with an acceptable complication rate in a variety of low-resource settings around the globe. The SHC facilitates early mobilization while maintaining acceptable fracture reduction until union. Caution should be exercised in older patients with poor bone quality.

## Conflict of interest

Dr. Zirkle reports that he is founder and president of SIGN Fracture Care International. Dr. Schlechter reports others from Arthrex, Inc., Naples, FL, outside the submitted work. Dr. Roth, Dr. Goldman, Mr. Ibrahim, and Dr. Shearer have nothing to disclose.
